# Microglial Imaging in Alzheimer’s Disease and Its Relationship to Brain Amyloid: A Human ^18^F-GE180 PET Study

**DOI:** 10.3233/JAD-230631

**Published:** 2023-12-06

**Authors:** Zhengshi Yang, Sarah J. Banks, Aaron R. Ritter, Jeffrey L. Cummings, Karthik Sreenivasan, Jefferson W. Kinney, Jessica K. Caldwell, Christina G. Wong, Justin B. Miller, Dietmar Cordes

**Affiliations:** a Cleveland Clinic Lou Ruvo Center for Brain Health, Las Vegas, NV, USA; b Department of Brain Health, University of Nevada Las Vegas, Las Vegas, NV, USA; c University of California San Diego, San Diego, CA, USA; d Hoag’s Pickup Family Neurosciences Institute, Newport Beach, CA, USA; e Chambers-Grundy Center for Transformative Neuroscience, Pam Quirk Brain Health and Biomarker Laboratory, Department of Brain Health, School of Integrated Health Sciences, University of Nevada Las Vegas (UNLV), Las Vegas, NV, USA; f Department of Psychology and Neuroscience, University of Colorado, Boulder, CO, USA

**Keywords:** Alzheimer’s disease, amyloid, ^18^F-GE180, neuroinflammation, translocator protein

## Abstract

**Background:**

Emerging evidence suggests a potential causal role of neuroinflammation in Alzheimer’s disease (AD). Using positron emission tomography (PET) to image overexpressed 18 kDA translocator protein (TSPO) by activated microglia has gained increasing interest. The uptake of ^18^F-GE180 TSPO PET was observed to co-localize with inflammatory markers and have a two-stage association with amyloid PET in mice. Very few studies evaluated the diagnostic power of ^18^F-GE180 PET in AD population and its interpretation in human remains controversial about whether it is a marker of microglial activation or merely reflects disrupted blood-brain barrier integrity in humans.

**Objective:**

The goal of this study was to study human GE180 from the perspective of the previous animal observations.

**Methods:**

With data from twenty-four participants having ^18^F-GE180 and ^18^F-AV45 PET scans, we evaluated the group differences of ^18^F-GE180 uptake between participants with and without cognitive impairment. An association analysis of ^18^F-GE180 and ^18^F-AV45 was then conducted to test if the relationship in humans is consistent with the two-stage association in AD mouse model.

**Results:**

Elevated ^18^F-GE180 was observed in participants with cognitive impairment compared to those with normal cognition. No regions showed reduced ^18^F-GE180 uptake. Consistent with mouse model, a two-stage association between ^18^F-GE180 and ^18^F-AV45 was observed.

**Conclusions:**

^18^F-GE180 PET imaging showed promising utility in detecting pathological alterations in a symptomatic AD population. Consistent two-stage association between ^18^F-GE180 and amyloid PET in human and mouse suggested that ^18^F-GE180 uptake in human might be considerably influenced by microglial activation.

## INTRODUCTION

Therapeutic interventions targeting reduction of brain amyloid-β protein (Aβ) in Alzheimer’s disease (AD) have shown benefits in clinical trials; however, the mechanistic role of Aβ plaques in AD pathogenesis remains controversial. Emerging evidence suggests that alternative but correlated disease pathways and their interaction with brain amyloid pathology could be crucial for understanding the pathological mechanisms of AD at early stages of the disease. Evidence from genetic, autopsy, animal model, and proteomic studies suggest that neuroinflammation is a key aspect of the pathophysiology of AD [1–4].

The activated microglia in the AD-associated inflammatory response are believed to be beneficial for clearance of accumulating toxic Aβ oligomers/plaques and restoring tissue homeostasis in the early stages of AD [5]. However, the neurotoxic substance released by chronic microglial activation could cause neuronal damage and neurodegeneration. In both autopsy studies and transgenic AD mouse models, Aβ plaques are surrounded by activated microglia [6]. These findings suggest that an individual with more aggregated amyloid deposits is more likely to have greater microglial activity. Assessing microglial activation and testing its relationship to brain amyloid burden in living human patients is challenging. Brain inflammation *in vivo* has been studied with positron emission tomography (PET) using radiotracers binding to the 18 kDA translocator protein (TSPO), one of the very few proteins in the central nervous system altered in neuroinflammation [7]. Various radiotracers, including ^11^C-PK-11195, ^11^C-AC5216, ^18^F-DPA-714, and ^18^F-GE180, have been developed to bind with TSPO [8–11]. The binding affinity to TSPO and the ratio of tracers crossing the blood-brain barrier (BBB) are the major factors limiting the interpretation of radiotracers binding to microglial activation.

The recently developed ^18^F-GE180 radiotracer was demonstrated to have higher binding affinity to TSPO than the ^11^C-PK-11195 in several mouse models of neuroinflammation [12–14]. By collecting ^18^F-GE180 microglial PET imaging and Pittsburgh compound B (^11^C-PiB) amyloid PET imaging data in the APP23 mouse model of AD, transgenic mice was found to have higher ^18^F-GE180 binding than wild-type mice [15]. In addition, a two-stage association between ^18^F-GE180 and amyloid PET was observed, namely ^18^F-GE180 binding initially increases with accumulated brain amyloid, but the signal plateaus despite continuing amyloidosis. ^18^F-GE180 binding in transgenic mice co-localized with the immunohistochemistry staining of ionized calcium-binding adaptor molecule 1 (Iba1), an inflammation marker, indicating that ^18^F-GE180 tracer could image the elevated microglial activation in transgenic mice, which is consistent with the observations from other animal studies [13, 14, 16–18]. This evidence collectively supported a signature two-stage association between microglial activation and amyloid in AD mouse model.

However, whether the ^18^F-GE180 signal in human brain is substantially derived from microglial activation or merely reflects the integrity of the BBB is unresolved. BBB permeability could vary with species [19]. ^18^F-GE180 has a good affinity for TSPO, but its uptake in the human brain is observed to be lower than another TSPO tracer ^11^C-PBR28 [20]. Therefore, the promising results from AD mice might not necessarily predict the performance in human patients. The required immunohistochemical staining technique used in mice to illustrate the biological interpretation of ^18^F-GE180 uptake are not feasible for living human subjects.

Reduced microglial metabolism was reported to be associated with decreased ^18^F-GE180 uptake in both mice and human, suggesting that the ^18^F-GE180 uptake in human might be beyond BBB permeability [21]. If the two-stage association between microglial activation and amyloid observed in mice could be replicated with human ^18^F-GE180 and amyloid PET data, it could further serve as an additional piece of evidence supporting that elevated ^18^F-GE180 uptake in human brain might be substantially derived from abnormal microglial activation, instead of merely driven by disrupted BBB integrity.

In this study, we conducted the analysis with ^18^F-GE180 PET and ^18^F-AV45 amyloid PET imaging data from a cohort of elderly participants. We hypothesized that the cognitively impaired (CI) participants have higher ^18^F-GE180 uptake than the cognitively unimpaired (CU) individuals, and that the association between ^18^F-GE180 and ^18^F-AV45 is consistent with the two-stage association observed with the AD mouse model. To test our hypothesis, we first compared the differences between participants with and without cognitive impairment, then analyzed the association between these two PET modalities.

## MATERIALS AND METHODS

### Subjects

The data used in this study were collected from the Center for Neurodegeneration and Translational Neuroscience (CNTN, https://nevadacntn.org/). All participants were recruited at Cleveland Clinic Lou Ruvo Center for Brain Health (CCLRCBH), Las Vegas, Nevada. The study was approved by Cleveland Clinic Institutional Review Board and all participants gave written, informed consent. Only the participants with mixed or high affinity binding affinity to TSPO as determined by a single nucleotide polymorphism in the TSPO gene [22] were recruited for ^18^F-GE180 PET imaging. Twenty-four participants, consisting of 12 CU participants and 12 CI participants diagnosed clinically with mild cognitive impairment or mild dementia, were included. Diagnosis was based on the National Institute on Aging/Alzheimer’s Association (NIA/AA) clinical criteria [23, 24] and supported by assessment with the Montreal Cognitive Assessment (MoCA; shown in Table 1) and other neuropsychological assessments [25]. All 24 participants had ^18^F-GE180 PET and T1 structural magnetic resonance imaging available. All participants except one had ^18^F-AV45 PET scan available. Participants exhibited a broad range of amyloid standardized uptake value ratios (SUVRs), allowing study of the association between ^18^F-GE180 PET and brain amyloid burden. Demographic characteristics of the participants included in the study are summarized in Table 1.

**Table 1:** Demographic characteristics of the study participants

	CU	CI	*p*
Age, y (SD)	70.75 (7.48)	74.58 (6.23)	0.19
Sex, M/F	06/06	07/05	0.68
Amyloid positivity	1/11/0	8/3/1	<0.001
(+/–/NA)
MoCA (SD)	27.08 (2.07)	21.83 (4.06)	<0.001
Weight, kg (SD)	78.4 (13.0)	78.0 (18.6)	0.95
Education, y (SD)	15.25 (2.05)	15.83 (2.59)	0.55

CU, cognitively unimpaired; CI, cognitively impaired; F, female; M, male; kg, kilogram; MoCA, Montreal Cognitive Assessment; SD, standard deviation; y, years; NA, not available.

### ^18^F-AV45 and ^18^F-GE180 PET acquisition

Amyloid PET data were acquired from enrolled subjects after injection of 370 MBq (±10%) of ^18^F-AV45 (florbetapir). PET images were acquired for 20 min at 50–90 min after injection. The images were reconstructed using three-dimensional ordered-subset expectation maximization to an image size 256×256 matrix, slice thickness of 2.0 mm, and post reconstruction Gaussian filter of 3 mm.

CCLRBH had the investigational new drug (IND) approval from United States Food and Drug Administration to use ^18^F-GE180 radiotracers for human PET imaging. ^18^F-GE180 PET imaging was acquired from enrolled participants after injection of 185 MBq (±10%) of ^18^F-GE180. Following the protocols suggested by the vendor, the PET data were collected for 30 min beginning 75 min after injection. The images were reconstructed using three-dimensional ordered-subset expectation maximization to an image size 256×256 matrix and slice thickness of 2.0 mm.

### Imaging analysis

The same analysis pipeline was used for both ^18^F-AV45 and ^18^F-GE180. Briefly, PET images were coregistered to their T1 structural image. The T1 images were input to FreeSurfer 6.0 (https://surfer.nmr.mgh.harvard.edu/) for anatomical segmentation and regional labelling. Regional SUVRs were computed by averaging the signal from gray matter voxels in each region and then normalized to the mean signal of the whole cerebellum. Then the mean SUVRs of four brain areas, including frontal, anterior/posterior cingulate, lateral parietal, and lateral temporal, were computed. The regions in each brain area can be found in Landau et al. [26]. The composite SUVR was calculated by averaging across these four brain areas. The composite SUVR from ^18^F-AV45 PET represents the overall assessment of brain amyloid, which is used in a binary approach to determine brain amyloid positivity with the cutoff of 1.11. Among the twelve CI participants, eight individuals were determined to be amyloid positive, three individuals were amyloid negative, and one individual had no ^18^F-AV45 amyloid PET scan. As to the twelve CU participants, only one subject was amyloid positive and all rest 11 subjects were amyloid negative.

### Statistical analysis

While cerebellum is widely used as the reference region for ^18^F-AV45 PET, there is no consensus about the reference region for ^18^F-GE180 PET. Therefore, we first used 2-sample *t*-tests to compare the mean signal for the cerebellum between CI and CU groups to validate its usage as the reference region for ^18^F-GE180 PET. The ^18^F-GE180 signal intensity at cerebellum did not show a difference between the CI and CU group (see Fig. 2a and Results), indicating that it is appropriate to use cerebellum as the reference region and the differences of composite and regional ^18^F-GE180 SUVR are not driven by the discrepancy of cerebellar signal between groups. The overall between-group difference of the ^18^F-GE180 signal was evaluated by using the composite SUVR, followed by a set of region-wise comparisons of SUVRs from 39 cortical and subcortical regions defined by FreeSurfer (left- and right-hemisphere combined; list of regions can be found in the Supplementary Table 1). The false discovery rate (FDR) was used to correct for multiple comparisons. The differences of the ^18^F-AV45 composite SUVR between CU and CI group were also calculated. Since brain amyloid accumulates widely in the cortex, we did not carry out a region-wise comparison of ^18^F-AV45. With the observation of nonlinear association between ^18^F-AV45 and ^18^F-GE180, a restricted cubic spline regression was used to fit the curve with the corresponding SUVRs from the four brain areas described above (frontal, anterior/posterior cingulate, lateral parietal and lateral temporal). A bootstrapping technique was used to obtain the 95% confidence interval of the fitting curve with 1000 iterations. Then separate linear fitting was conducted to evaluate the association with ^18^F-AV45 SUVR below or above 1.15. This value was determined by the “elbow” point of the nonlinear fitting curve, indicating the transition point of the association between ^18^F-GE180 and ^18^F-AV45, which is irrelevant to amyloid positivity.

## RESULTS

**Fig. 1:**
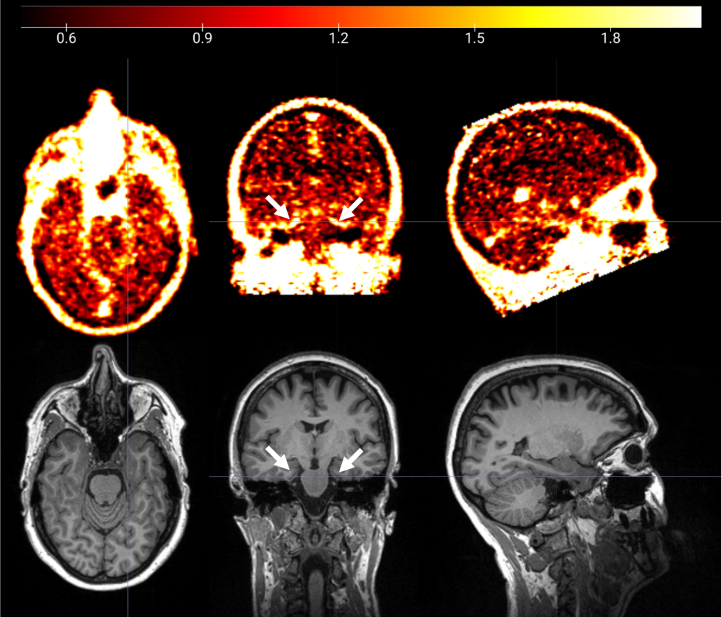
^18^F-GE180 PET SUVR image (upper panel) and T1 structural MRI image (lower panel) from a cognitively impaired participant. Elevated ^18^F-GE180 uptake following the contour of entorhinal cortex and parahippocampal gyrus (pointed by white arrows) was observed.

**Fig. 2:**
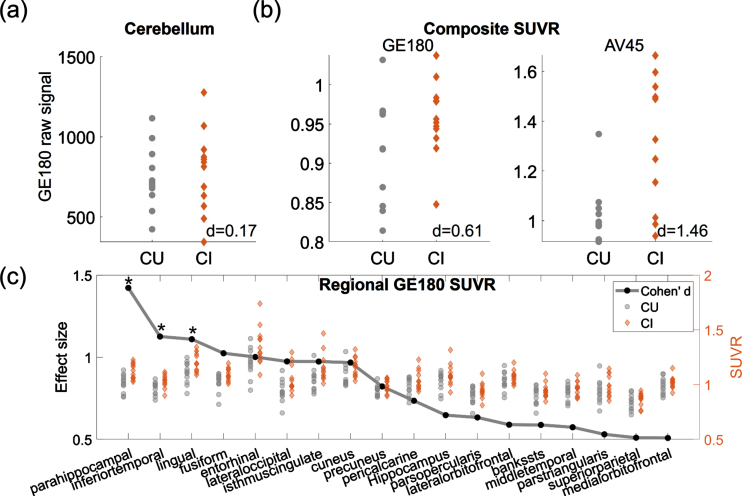
Group comparison between CU and CI participants. (a) Group comparison of the mean intensity of raw ^18^F-GE180 signal in cerebellum. (b) Group comparison of ^18^F-GE180 and ^18^F-AV45 composite SUVRs. (c) Group comparison of regional ^18^F-GE180 SUVRs. Only the regions with at least moderate effect sizes (Cohen’s d > = 0.5) are shown in the plot. The effect sizes are marked as black dots. Asterisks (*) indicate the regions showing significant group differences after FDR correction. Gray dots and brown diamonds indicate the regional ^18^F-GE180 SUVRs for CU (cognitively unimpaired) and CI (cognitively impaired) participants, respectively.

The ^18^F-GE180 SUVR image from one CI participant is shown on the upper panel of Fig. 1, and the co-registered T1 structural image is presented on the lower panel. Elevated signal intensity following the contour of parahippocampal gyrus (pointed by white arrows) was observed. The group comparison of the original ^18^F-GE180 signal at cerebellum did not show a difference between the CI and CU group (Fig. 2a; *p* > 0.05 and effect size d = 0.17). The CI group had higher composite SUVR than the CU group with moderate effect for ^18^F-GE180 (Fig. 2b; Cohen’s d = 0.61) and very large effect for ^18^F-AV45 (Fig. 2b; Cohen’s d = 1.46), although the difference for ^18^F-GE180 composite SUVR did not reach the significance level due to limited sample size.

The regions having group differences with at least moderate effect sizes (Cohen’s d > = 0.5) are shown in Fig. 2c, with black dots representing the effect sizes and the gray dots and brown diamonds indicating the regional SUVRs for CU and CI participants, respectively. Higher ^18^F-GE180 SUVR in CI group was observed mainly in the parietal-temporal area. The group differences at parahippocampal, inferior temporal gyrus and lingual gyrus were significant after FDR correction (corrected *p* value <0.05). The most substantial regional difference was observed at parahippocampal gyrus with a very large effect (Cohen’s d = 1.42). No regions in the CI group showed lower ^18^F-GE180 compared to CU group. The complete list of regional comparisons can be found in Supplementary Table 1.

**Fig. 3:**
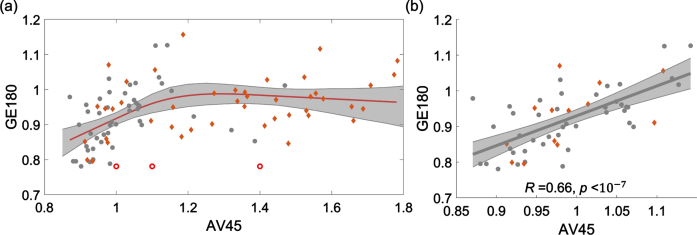
Scatter plot of ^18^F-AV45 versus ^18^F-GE180 SUVRs from four brain areas, including frontal, anterior/posterior cingulate, lateral parietal, and lateral temporal. (a) A restricted cubic spline regression is conducted with the full range of AV45 SUVR. ^18^F-GE180 was positively correlated to ^18^F-AV45 when the ^18^F-AV45 was relatively low (approximately <1.15) and then became poorly correlated when ^18^F-AV45 was higher. (b) A linear fitting analysis was conducted in the lower ^18^F-AV45 range with SUVR <1.15. Similar trends were observed when the fitting was conducted for these four brain areas separately (Supplementary Figure 1).

^18^F-AV45 and ^18^F-GE180 PET data were demonstrated to have a nonlinear relationship, as shown in the scatter plot of SUVRs from four brain areas, including frontal, anterior/posterior cingulate, lateral parietal, and lateral temporal (Fig. 3a). When the ^18^F-AV45 was relatively low (approximately <1.15), ^18^F-GE180 was positively correlated to ^18^F-AV45. When ^18^F-AV45 was higher, ^18^F-GE180 became poorly correlated to ^18^F-AV45 and presented as a flat fitting curve in the figure. To evaluate the significance level of the positive association in the lower range of ^18^F-AV45, a separate linear fitting was conducted to evaluate the association within the range of ^18^F-AV45 SUVR <1.15, a significant association between ^18^F-AV45 and ^18^F-GE180 was observed with *R* = 0.66 (*R*-squared = 0.44) and *p* value <10^–7^. Such an association remained to be highly significant when we used linear mixed effect model to evaluate the association with brain area modelled as a random effect, suggesting that such an association was not due to multiple measures from each individual used in the analysis. The separate scatter plots for these four brain areas in the lower range are shown in Supplementary Figure 1, similar positive associations were consistently observed at these four brain areas.

## DISCUSSION

Our study assessed a disease cohort having both ^18^F-GE180 and ^18^F-AV45 PET imaging available to help assess diagnostic power of ^18^F-GE180 and its relationship with brain amyloid burden. Very few studies investigated the diagnostic power of ^18^F-GE180 PET and its association with brain amyloid pathology. There is ongoing controversy about whether elevated ^18^F-GE180 uptakes represents overexpressed TSPO or reflects BBB integrity. This study was intended to evaluate the capability of ^18^F-GE180 PET in detecting pathological alterations in patients with cognitive impairment and provide additional evidence to clarify its potential biological meaning in human brains.

By using the same analysis pipeline as ^18^F-AV45 amyloid PET imaging, a ^18^F-GE180 composite score representing the overall assessment of radiotracer uptake in the brain was derived. At the whole brain level, ^18^F-GE180 uptake was found to be moderately higher in participants with cognitive impairment compared to those without cognitive impairment, whereas the ^18^F-AV45 uptake achieved a much more substantial difference between groups.

Region-wise analysis showed that nearly all regions had higher ^18^F-GE180 uptake in the CI group, but the difference varied from very small (Cohen’s *d* < 0.1) to very large (Cohen’s *d* > 1.2), with the regions showing substantially elevated ^18^F-GE180 uptake mainly located in the parietal-temporal area, including middle temporal gyrus, parahippocampal, hippocampus, superior parietal lobule, and precuneus. The regions identified match the known topology of the microglial activation in neurodegenerative diseases [21, 27, 28]. Parahippocampal gyrus was observed to have the most striking group difference. A previous report with an independent cohort also demonstrated elevated ^18^F-GE180 uptake in patients with AD [28]. The regions identified in our study are implicated in studies of the regional neuropathological changes in AD, including brain atrophy, hypometabolism, and tau pathology [29–31].

The variability of regional differences suggests that a relatively spatially-focal pathological alteration was detected by ^18^F-GE180. In fact, microglial activation was demonstrated to propagate and colocalize with tau pathology in a Braak stage-like fashion [32, 33]. The regions showing elevated ^18^F-GE180 in CI groups, such as hippocampus and entorhinal cortex, are the area susceptible to tau pathology at the early Braak stage. Based on the effect sizes in the group comparison, the parahippocampal (posterior portion of parahippocampal gyrus) had larger difference than the entorhinal cortex (anterior portion of the parahippocampal gyrus) and the hippocampus, although they are functionally and anatomically close to each other. Possibly due to their anatomical positions, the mean magnitude and the between-subject variance of ^18^F-GE180 SUVRs differed substantially at these three regions, which might contribute to the discrepancy of group differences observed among them.

In contrast, both autopsy and amyloid PET imaging studies have shown that, consistent with our findings, amyloid-β accumulates in nearly all cortical regions early in the disease continuum [34, 35]. The limited spatial extension of abnormal ^18^F-GE180 uptake, in contrast to widespread amyloid accumulation observed with ^18^F-AV45 amyloid PET, may contribute to the smaller difference observed with the ^18^F-GE180 composite score compared to ^18^F-AV45 composite score.

Preclinical imaging studies showed that the ^18^F-GE180 PET ligand co-localized with immunohistochemical staining of an inflammatory marker [15], indicating the feasibility of ^18^F-GE180 in detecting neuroinflammation in mice. In human subjects, the biological meaning of ^18^F-GE180 PET is uncertain. BBB permeability varies across species [19] and the observations in mice regarding ^18^F-GE180 do not necessarily guide human conclusions. Given the low permeability of the BBB to ^18^F-GE180 in human PET, some suggested that ^18^F-GE180 PET signal mainly reflected the integrity of BBB and was not specific for TSPO [36–38]. However, ^18^F-GE180 showed promising results in detecting neuroinflammation in patients with multiple sclerosis [39] or gliomas [40]. In these disease conditions, areas with compromised BBB did not necessarily show significant ^18^F-GE180 uptake while increased uptake was found in regions without visible contrast enhancement in gadolinium-contrasted MRI image expected with BBB impairment [41]. In addition, one previous study showed that pharmacological depletion of microglia led to decreased ^18^F-GE180 signal in mice and the reduction of ^18^F-GE180 signal was positively associated with reduction of cerebral glucose uptake in both mice models and patients with neurodegenerative diseases [21], suggesting that microglial activation contributes, at least partially, to the signal observed in ^18^F-GE180 human PET imaging. In this study, we showed that ^18^F-GE180 was positively correlated with ^18^F-AV45 when ^18^F-AV45 was low and was poorly correlated when the ^18^F-AV45 SUVR was higher. This finding suggests that ^18^F-GE180 uptake initially increases with brain amyloid and then plateaus with continuing amyloidosis. Microglial activation was found to be prior to tau pathology [33], and amyloid pathology was followed by tau pathology in the amyloid cascade model [42]. The positive association between ^18^F-GE180 and ^18^F-AV45 observed in the study in the lower amyloid range is in line with this previous research. The poor association in the higher amyloid range could be due to the collective effect of more concurrent pathological alterations (e.g., atrophy) with disease progression. The two-stage association of human ^18^F-GE180 PET with amyloid PET was consistent with previous observations in preclinical study [15], where increased ^18^F-GE180 uptake was demonstrated to colocalize with an inflammatory marker through immunohistochemistry staining technology. An independent study [27] showed that ^18^F-GE180 SUVR was positively associated with cerebrospinal fluid soluble triggering receptor expressed on myeloid cells 2 (sTREM2), a fluid marker of activated microglia, when sTREM2 concentration was less than 15 ng/ml. These results collectively provide evidence suggesting that the ^18^F-GE180 uptake in human brain is more likely substantially derived from microglial activation, instead of merely a marker of BBB integrity. Collectively, the evidence of higher ^18^F-GE180 SUVRs in regions prone to tau pathology and its relevance with brain amyloid suggested the potential utility of ^18^F-GE180 to detect pathological changes since the early stage.

There are limitations with the study. First, although the consistent association of ^18^F-GE180 and ^18^F-AV45 in human and mice indirectly suggests that neuroinflammation leads to the elevated ^18^F-GE180 uptake in CI participants as observed in our study, more direct evidence to exclude or adjust for the influence of BBB breakdown would further validate the biological meaning of the findings. We do not have gadolinium-enhanced MRI or postmortem brains available to evaluate the potential influence of BBB breakdown. The serum level of S100b protein is an emerging candidate peripheral marker of BBB permeability [43], and testing its relevance to ^18^F-GE180 may be an alternative approach to investigate the influence of BBB breakdown on the PET data. Second, our study was conducted with cross-sectional data, longitudinal studies of ^18^F-GE180 in humans would be beneficial to further consolidate our findings. Third, the number of participants in this study was relatively small. All previous ^18^F-GE180 studies were reported with cohorts of comparable sizes [21, 27, 28]. The exploratory nature of the current study requires independent studies to confirm the findings. Forth, the ^18^F-GE180 PET scans were collected up to 2 years after ^18^F-AV45 PET scans. Amyloidosis in AD is a slow process and its accumulation lasts for more than decades before onset of cognitive syndromes [44], therefore, brain amyloid load was not expected to change substantially in the time gap. In addition, the time gap did not differ between CU and CI groups.

In summary, our study demonstrated the potential utility of *in vivo* ^18^F-GE180 PET in detecting the pathological alterations in patients with cognitive impairment and provides evidence supporting that ^18^F-GE180 uptake in humans might be substantially influenced by an innate immune response, instead of merely a marker of disrupted BBB integrity, although more evidence is required to further examine the robustness of ^18^F-GE180 in assessing inflammation in human subjects.

## Supplementary Material

Supplementary Material

## Data Availability

The data supporting the findings of this study are available through data request at https://nevadacntn.org/.
