# Supplementary material for: Microglial Imaging in Alzheimer’s Disease and Its Relationship to Brain Amyloid: A Human 18F-GE180 PET Study
Source: J Alzheimers Dis. 2023 Dec 6;96(4):1505–14. doi: 10.3233/JAD-230631 (PMC10894577; doi:10.3233/JAD-230631)

Supplementary Material

Microglial Imaging in Alzheimer's Disease and Its Relationship to Brain Amyloid: A Human ^{18}F-GE180 PET Study

Supplementary Table 1. ROI comparison

Region	Cohen's d		
parahippocampal	1.42	superiortemporal	0.48
inferiortemporal	1.13	rostralanteriorcingulate	0.44
lingual	1.11	inferiorparietal	0.43
fusiform	1.02	Amygdala	0.41
entorhinal	1.00	precentral	0.40
lateraloccipital	0.97	transversetemporal	0.34
isthmuscingulate	0.97	insula	0.32
cuneus	0.97	parsorbitalis	0.31
precuneus	0.82	posteriorcingulate	0.29
pericalcarine	0.74	caudalanteriorcingulate	0.27
Hippocampus	0.65	rostralmiddlefrontal	0.23
parsopercularis	0.63	supramarginal	0.22
lateralorbitofrontal	0.59	Putamen	0.19
bankssts	0.59	postcentral	0.18
middletemporal	0.57	superiorfrontal	0.08
parstriangularis	0.53	paracentral	0.07
superiorparietal	0.51	Pallidum	0.07
medialorbitofrontal	0.51	Thalamus	0.04
caudalmiddlefrontal	0.49	Caudate	0.00
		temporalpole	0.00

Supplementary Figure 1

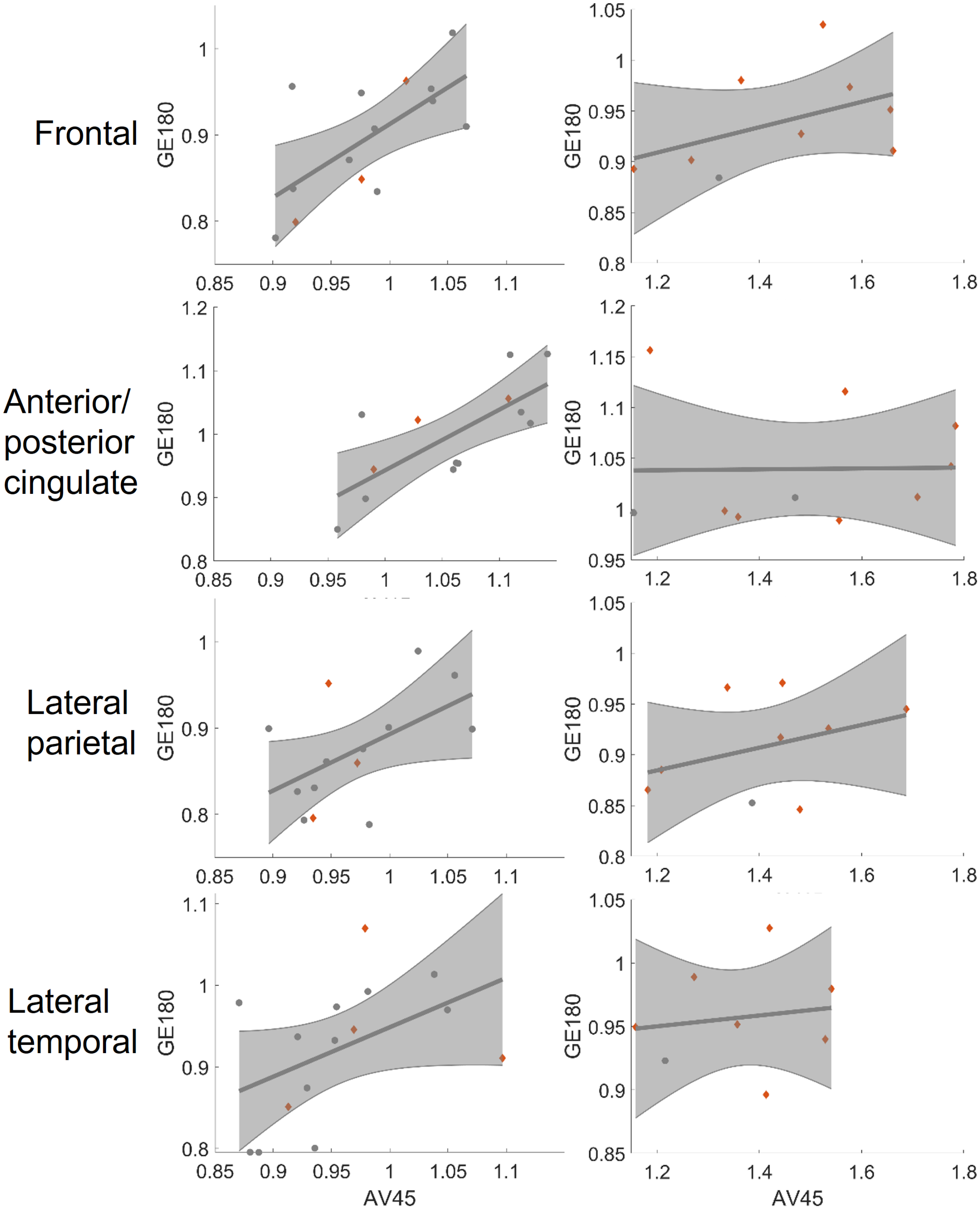

Supplement: Supplementary Material [file jad-96-jad230631-s001.pdf]
